# Flasks and Flasking

**Published:** 1883-01

**Authors:** 


### Flasks and Flasking.

[Read before the Detroit Dental Society by, E. C. MOORE, D.D.S., Dec. 4, 1882.]

GENTLEMEN:—In the lull or lack of other and possibly more important business, I would like to present for your inspection and criticism a modified form of brass flask for rubber work which answers my purpose better than anything I have ever used in the flask line, and while we are on the subject I will venture a few remarks in connection with flasks and flasking. It is conceded, I believe, that brass, taking all in all, is a superior metal to iron for flasks. While the metal is not as rigid or stiff as iron, the advantage of being more cleanly to handle and non-corrosive, more than compensates for its deficiency in rigidity; being non-corrosive makes it everlasting, and, from an economical point of view, the cheapest flask to buy, but I contend farther (that the way I have constructed this flask (that it is abundantly stiff for any legitimate purpose to which it should be subjected when anticipating a good piece of work, and this means (so far as the flask itself may be held responsible), "good joints in block teeth work." Of course the proper and necessary care being taken in packing the rubber and closing the flask there will not be force enough applied to in the least change the shape of the flask in any part. On inspection you will observe that the top plate or cover of the flask is well protected or braced by a good body of metal on the ring middle or second section coming well up under the projections on the top plate where the bolts pass through the point most likely, if any, to yield under pressure or force. The bolts are made of the best Swedish iron and are very tough, and cost about 3 cents each, and as another means of protection to the flask as well as saving the bolts, are provided with washers. These I have found to be of great advantage other than that of protection of flasks and bolts.

Imagination has so worked upon the minds of some as to bring forth the remark that the flask is not large enough; they can be convinced to the contrary on trial. If we were going to roll up a sheet of rubber in a ball and place in the centre of our case and screw together regardless, then the flask is not large enough, the bolts are not large or strong enough, the plaster is not strong enough, and the teeth should be of adamant and the joints soldered together with Dr. Land's cross-pins, top and bottom. I may say in this connection that last Friday afternoon I framed, arranged, waxed, invested and vulcanized an upper set, and one, too, where the front blocks came close on to the cast. The joints came out nice and clean; no discoloration. They would not have been better if I had waited a week, or had invested and vulcanized in a barrel of plaster, showing that neither time or quantity of plaster is necessary in the production of good work, but rather it is the result of care and quality of plaster. I have never experienced the least inconvenience in this particular. I cannot see the advantage of more plaster; it is simply a superfluity, and in support of this I may say that I have approximated (although I have not quite reached 6,000 plates) the average of a plate a day since a year ago the first of November, and I have not in that time or number found a single case that I could not with perfect ease and satisfaction get into this flask. I have, however, seen cases that would not go into any ordinary flask; these are monstrosities and, of course, are to be excepted. Mechanically speaking, it is a mistake in having a large flask, to get in the requisite amount of plaster for a thorough investing is all that is necessary; the smaller the flask the better control one has over the work, or, in other words, the resistance to be overcome (the molding of the rubber into form of plate) is not so far from the application of force or power, the distance being diminished the leverage is proportionately increased, the strain is also diminished both on flask and bolts. I may say that I rarely break a bolt in closing the flask or have rubber make its appearance on the outside of the flask; the bolts are absolutely corroded out and the thread will no longer hold the nuts.

Now a word about flasking; and to go about this properly we will go back to the preparation of the impression. As soon as the impression is taken and hardened a few minutes, (I have in view now more particularly full sets), with a suitable knife shave off the rim of the impression to a point very near where you think or want your plate to extend in the mouth, including the bevel a little in or down. This will assist in separating the impression from the model, and will present this appearance on the model. A continuous line and shoulder from heel to heel about $\frac{1}{16}$ of an inch wide, and in waxing or modeling the case wax right down to this shoulder clean cut all around, and then when you come to invest, your model should be of sufficient thickness to bring this line and shoulder on a line, or a little above, the upper edge of the flask, or the joint between the parts. This will insure you the strongest possible edge to your plaster and leave the model entirely above the surface of the surrounding investing plaster where you can get at it to varnish or tin over, and will easily separate during the process of packing for inspection, which, to my mind, is absolutely necessary if you would secure a good fit, good joints, and clean plate. Regarding the completion of the flasking process no other extraordinary precautions are necessary, excepting that great care should be taken not to allow the plaster to extend anywhere on the bearings of the flask so that when closed all parts will come perfectly together, brass to brass, clean home, and the surplus rubber, if any, will be contained in gates cut in the investing plaster. In this way you will retain the same articulation you had on your articulator, which, if correct, will require little or no grinding of the teeth when placed in the mouth.

I have only one more suggestion to make in connection with this subject, and to those who use the brass flask; don't use a hammer for knocking out the plaster, but a wood, horn, or rawhide mallet.

